# One Year Outcomes Following Transplantation with COVID-19-Positive Donor Hearts: A National Database Cohort Study

**DOI:** 10.3390/jcdd11020046

**Published:** 2024-01-31

**Authors:** Stanley B. Wolfe, Ruby Singh, Dane C. Paneitz, Seyed Alireza Rabi, Chijioke C. Chukwudi, Richa Asija, Eriberto Michel, Asvin M. Ganapathi, Asishana A. Osho

**Affiliations:** 1 Division of Cardiac Surgery, Massachusetts General Hospital, Harvard Medical School, Boston, MA 02114, USA; 2 Department of Surgery, Allegheny General Hospital, Pittsburgh, PA 15212, USA; 3 Department of Surgery, Johns Hopkins Hospital, Baltimore, MD 21287, USA; 4 Department of Surgery, Community Memorial Hospital, Ventura, CA 93003, USA; 5 Division of Cardiac Surgery, Ohio State University Wexner Medical Center, Columbus, OH 43210, USA

**Keywords:** coronavirus disease 2019, COVID-19 donor hearts, heart transplant, transplant outcomes, recipient survival, donor selection

## Abstract

The current understanding of the safety of heart transplantation from COVID-19+ donors is uncertain. Preliminary studies suggest that heart transplants from these donors may be feasible. We analyzed 1-year outcomes in COVID-19+ donor heart recipients using 1:3 propensity matching. The OPTN database was queried for adult heart transplant recipients between 1 January 2020 and 30 September 2022. COVID-19+ donors were defined as those who tested positive on NATs or antigen tests within 21 days prior to procurement. Multiorgan transplants, retransplants, donors without COVID-19 testing, and recipients allocated under the old heart allocation system were excluded. A total of 7211 heart transplant recipients met the inclusion criteria, including 316 COVID-19+ donor heart recipients. Further, 290 COVID-19+ donor heart recipients were matched to 870 COVID-19− donor heart recipients. Survival was similar between the groups at 30 days (*p* = 0.46), 6 months (*p* = 0.17), and 1 year (*p* = 0.07). Recipients from COVID-19+ donors in the matched cohort were less likely to experience postoperative acute rejection prior to discharge (*p* = 0.01). National COVID-19+ donor heart usage varied by region: region 11 transplanted the most COVID-19+ hearts (15.8%), and region 6 transplanted the fewest (3.2%). Our findings indicate that COVID-19+ heart transplantation can be performed with safe early outcomes. Further analyses are needed to determine if long-term outcomes are equivalent between groups.

## 1. Introduction

In the early period of the coronavirus disease 2019 (COVID-19) pandemic, transplant centers were hesitant to utilize organs from COVID-19-positive (COVID-19+) donors for several reasons, including the clinically profound systemic inflammatory response exhibited by patients and the unknown transmissibility between the organ donor and recipient [1]. The International Society of Heart and Lung Transplantation published a guidance statement recommending that all donors should be screened for COVID-19 and that their organs be declined for transplantation if found to be Severe Acute Respiratory Syndrome Coronavirus 2 (SARS-CoV-2) polymerase chain reaction (PCR)-positive [2]. Meanwhile, the American Society of Transplantation published a statement recommending the use of organs from donors with a history of COVID-19 only if they are at least 21 days from the onset of symptoms and are SARS-CoV-2 PCR-negative [3,4].

As the pandemic continued, transplant centers became more willing to accept the risk of transplanting organs from COVID-19+ donors. While the transmission of COVID-19 between donors and recipients may not be a major concern, the lasting effects of COVID-19 on the donor heart and its performance in the recipient have yet to be assessed. The severe inflammatory response induced by SARS-CoV-2 results in the release of cytokines that can result in damage of endothelial cells and cardiac myocytes [5,6,7]. It is not known how the combination of SARS-CoV-2 exposure in addition to allograft ischemia during the procurement process may affect the performance of cardiac allografts. Studies on the 90-day outcomes of utilization of COVID-19+ donor hearts have reported similar survival in recipients from COVID-19-positive donors as compared to COVID-19-negative donors [8,9].

Although the lack of transmission of COVID-19 between donors and recipients and similar early outcomes of COVID-19+ and COVID-19− donor heart recipients is encouraging, it is essential to explore longer term outcomes. This study aims to analyze the 1-year outcomes in recipients of hearts from COVID-19+ donors.

## 2. Materials and Methods

### 2.1. Study Population

The study protocol was approved by the Mass General Brigham Institutional Review Board; individual consent was not required. A retrospective cohort study with propensity score matching was performed to compare outcomes in recipients of COVID-19+ donor hearts with outcomes in recipients of COVID-19− donor hearts using the Organ Procurement and Transplantation Network (OPTN) data as of March 2023. The Deceased Donor and Thoracic Data databases were queried for all cardiac transplant recipients from the pronouncement of the COVID-19 pandemic in January 2020 to 30 September 2022, 6 months prior to the date of the data to account for the differences in follow-up reporting by centers to the OPTN. Additionally, data on the specimen type used for COVID-19 testing and the number of days since testing positive for deceased donors prior to transplant were also included in the analysis. Pediatric recipients, multiorgan transplants, retransplants, recipients classified with the old heart allocation system used prior to 2018, recipients with missing patient status, and donors without COVID-19 test results or with indeterminate or pending test results were excluded.

Data were filtered to include donors who were tested for COVID-19. Recipients corresponding to these donors were matched by Donor ID. Donors who tested positive for COVID-19 with a nucleic acid amplification test (NAT) or antigen test from an upper respiratory or lower respiratory sample collected within 21 days of organ donation were considered to have a history of being COVID-19+. A sub-analysis was performed to explore outcomes in recipients who received cardiac allografts from donors with an active COVID-19 infection at the time of organ donation. Active COVID-19 was defined as organ donation from individuals who tested positive for SARS-CoV-2 on the final NAT or antigen testing before procurement. They were compared to a non-active group comprising recipients of hearts from donors with a negative final test before procurement (COVID-19−) as well as donors who had a history of COVID-19 infection but who were not acutely ill.

### 2.2. Statistical Analysis

Continuous variables are presented as “median (interquartile range)”, and categorical variables as “frequency (percentage)”. Continuous variables were assessed for normality using the Kolmogorov–Smirnov test and the association of non-normal variables among the two groups was assessed using the Wilcoxon rank-sum test. Categorical variables were assessed using the Chi-squared test or Fisher’s exact test for cell frequencies less than 5. The baseline variable of the donor–recipient predicted heart mass ratio was calculated as a categorical variable with a cutoff of <0.86 being considered undersized [10].

The primary outcome of interest was post-transplant 1-year all-cause mortality. Secondary outcomes included all-cause mortality over 30 days and 6 months post-transplant, acute rejection prior to discharge, length of stay at index hospitalization, and other postoperative outcomes of stroke, permanent pacemaker placement, and dialysis requirement prior to discharge from index hospitalization. Censored outcomes were assessed using Kaplan–Meier curves. For time-to-event outcomes, the proportional hazards (PH) assumption was tested using the Supremum test, and univariable Cox regression models were used to assess the risk of an event. The associations between COVID-19+ donor heart recipients and postoperative outcomes were assessed using univariable linear and logistic regression models. Finally, national usage and organ utilization rates of COVID-19+ and COVID-19− donor hearts were assessed by region.

Predictor variables with greater than 20% missing data were excluded from the study analysis. Statistical significance was defined as a two-sided *p* value < 0.05. Statistical analysis was performed using SAS 9.4 (SAS Institute Inc., Cary, NC, USA) and Stata 17 (StataCorp LLC, College Station, TX, USA). A map to illustrate the national trends was created using MapChart [11]. 

### 2.3. Propensity Matching

Propensity score calculation, as described by Austin [12], Rosenbaum, and Rubin [13] and utilized in cardiovascular research by D’Agostino [14], was used to match COVID-19+ heart recipients with COVID-19− heart recipients in a 1:3 ratio. The differences in patient, donor, and transplant characteristics between cohorts were controlled with this method. In this analysis, the propensity score estimates the probability of receiving a heart from a COVID-19+ donor, conditional on the pre-transplant recipient and donor characteristics. Propensity score matching was performed using a digit-based greedy matching algorithm by Parsons [15]. The score was calculated using a non-parsimonious logistic regression model using a pre-defined set of variables. Recipient variables used in the baseline model were age, gender, body mass index, history of diabetes, dialysis prior to transplant, status at transplant, baseline diagnosis, on ventilator at transplant, on inotropes at transplant, on extracorporeal membrane oxygenation (ECMO) at transplant, history of sternotomy, waiting list status, and mean pulmonary artery pressure. The donor variables used were age, history of smoking, cocaine use, cause of death, donation after circulatory death, predicted heart mass ratio < 0.86, recipient–donor sex mismatch, and ischemic time. The balance between the matched cohorts was assessed using standardized mean differences, with values below 0.2 indicating negligible differences in characteristics.

## 3. Results

### 3.1. Study Population Characteristics

**Figure 1:**
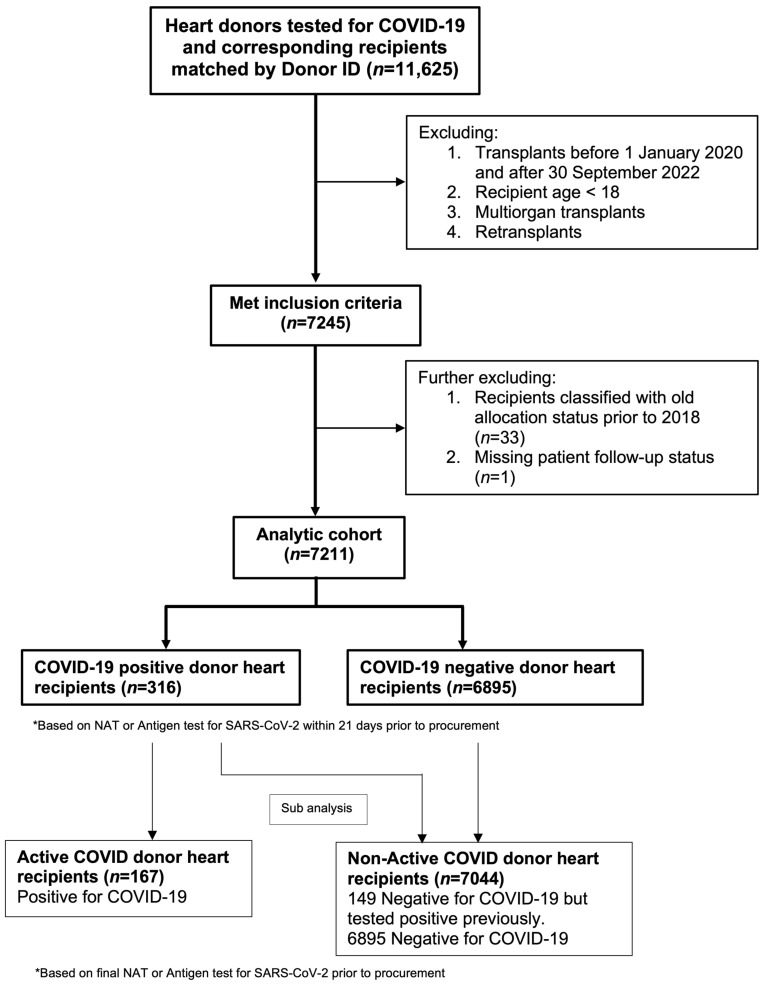
Flowchart depicting the inclusion criteria for patient selection using the Organ Procurement and Transplantation Network database.

A total of 7245 heart recipients met the inclusion criteria. Thirty-three recipients classified using the old recipient heart allocation system prior to 2018 and one recipient with a missing patient follow-up status was excluded from the analysis. The analytic cohort comprised 7211 patients who underwent heart transplantation, including 316 (4.4%) COVID-19+ donor heart recipients and 6895 (95.6%) COVID-19− donor heart recipients (Figure 1). Among the COVID-19+ donors, upper respiratory tract specimens were tested for COVID-19 in 201 (63.6%) donors and lower respiratory tract specimens were tested in 113 (35.8%) donors. The median time from testing to donation for these donors was 3 (IQR: 2–4) days.

Among the recipient characteristics, COVID-19+ donor heart recipients were more likely to have blood group O when compared to COVID-19− donor heart recipients (48.4% vs. 40.7%, *p* = 0.02). COVID-19+ donors were younger than COVID-19− donors (30 years vs. 32 years, *p* = 0.002), and more likely to be male (78.5% vs. 72.3%, *p* = 0.02). Additionally, the COVID-19+ donors were less likely to have died due to stroke (7.3% vs. 12.3%, *p* = 0.02). There were no other significant differences between the COVID-19+ and COVID-19− groups in terms of donor and recipient pre-transplant characteristics (Table 1).

### 3.2. Outcomes in Unmatched Population

**Figure 2:**
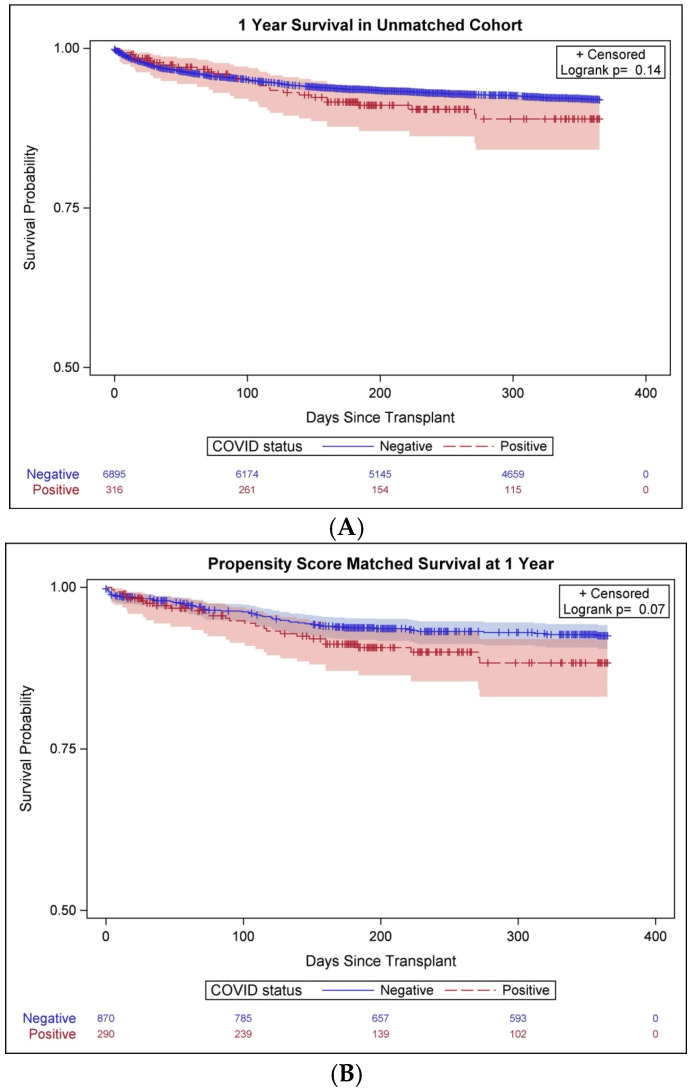
Kaplan–Meier curves showing similar 1-year survival among recipients of hearts from coronavirus disease 2019 (COVID-19)-positive and COVID-19-negative donors in (**A**) the unmatched cohort and (**B**) the propensity-matched cohort.

The primary outcome of 1-year all-cause mortality was similar between the heart recipients of COVID-19+ and COVID-19− donors (HR = 1.3, 95% CI: 0.91–1.95, *p* = 0.14, Figure 2A). There was also no difference in short-term all-cause mortality at 30 days (HR = 0.58, 95% CI: 0.38–1.72, *p* = 0.58) and 6 months between the two groups (HR = 1.28, 95% CI: 0.85–1.92, *p* = 0.25). The median follow-up time for the overall cohort was 12 (IQR: 6–23) months.

Among COVID-19+ donors, there was a 38% lower odds of experiencing acute rejection prior to discharge from the hospital when compared to COVID-19− donor heart recipients (OR = 0.62, 95% CI: 0.44–0.88, *p* = 0.007). Similarly, the odds of incidence of stroke were lower among COVID-19+ donor heart recipients (OR = 0.40, 95% CI: 0.16–0.98, *p* = 0.04). There was no difference in index hospitalization length of stay (*p* = 0.26), postoperative placement of permanent pacemaker (*p* = 0.10), and postoperative dialysis requirement (*p* = 0.21) between the two groups.

### 3.3. Outcomes in the Matched Population

In the matched cohort (*n* = 1160), 870 COVID-19− donor heart recipients were matched to 290 COVID-19+ donor heart recipients. Differences seen in recipient ABO grouping, donor age, donor sex, and donor cause of death in the unmatched cohort were no longer significant, and the groups were comparable (Table 2).

Like the unmatched cohort, there was no difference in 1-year all-cause mortality among COVID-19+ and COVID-19− donor heart recipients (HR = 1.53, 95% CI: 0.97–2.41, *p* = 0.07, Figure 2B). Mortality rates in recipients of COVID-19+ donor hearts at 30 days (HR = 1.40, 95% CI: 0.57–3.43, *p* = 0.46) and 6 months (HR = 1.41, 95% CI: 0.86–2.30, *p* = 0.17) were also comparable among the two groups.

The matched cohort continued to have a significant difference in acute rejection in COVID-19+ heart recipients with lower odds of experiencing acute rejection (OR = 0.61, 95% CI: 0.41–0.90, *p* = 0.01) compared to COVID-19− donor heart recipients. However, after matching, the incidence of stroke did not differ between the two groups (*p* = 0.11). Like in the unmatched cohort, there was no difference in other secondary outcomes of index hospitalization length of stay (*p* = 0.13), permanent pacemaker placement (*p* = 0.26), or dialysis requirement (*p* = 0.27) during index hospitalization among the two groups.

### 3.4. Sub-Analysis

A sub-analysis was performed comparing recipients of hearts from donors with active COVID-19 to recipients from donors with non-active COVID-19. The sub-analysis cohort consisted of 167/7211 (2.32%) recipients of hearts from donors an active COVID-19 infection at the time of procurement. The median time from testing to donation was 3 (IQR: 2–4) days in both groups. Recipient mortality rates at 1 year (*p* = 0.32), 6 months (*p* = 0.45), and 30 days (*p* = 0.80) were similar between the recipients of hearts procured from donors with active COVID-19, and those with hearts from donors with non-active COVID-19.

### 3.5. National Trends

**Figure 3:**
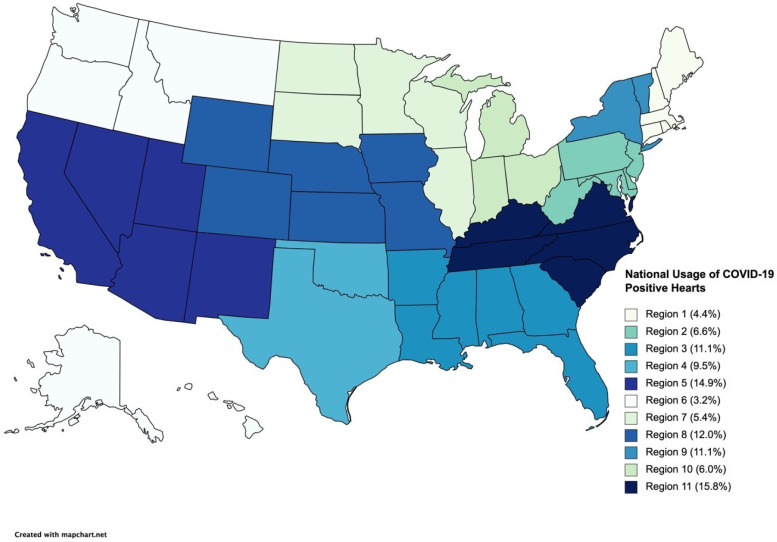
Map illustrating nationwide usage of hearts from coronavirus disease 2019 (COVID-19)-positive donors per Organ Procurement and Transplantation Network (OPTN) region.

Of the total national usage of COVID-19+ donor hearts (*n* = 316), region 11 transplanted the most COVID-19+ donor hearts [50/316 (15.8%)], while region 6 [10/316 (3.2%)] transplanted the fewest (Figure 3). The largest proportion of COVID-19+ donor heart transplantations per region was in region 8 [38/523 (7.3%)] and the smallest was in region 2 [21/665 (3.2%)].

## 4. Discussion

In this propensity-matched analysis of the Organ Procurement and Transplantation Network national database, we found no difference in 1 year, 30-day, or 6-month survival among heart transplant recipients who received COVID-19+ donor heart allografts compared to those who received COVID-19− donor heart allografts. We also conducted a sub-group analysis of donors with active COVID-19 at the time of organ procurement that notably did not demonstrate an association between active COVID-19 donor infection and worse recipient survival. Finally, we found that COVID-19+ donor heart recipients were less likely to experience acute rejection compared to COVID-19− donor heart recipients in both the matched and unmatched cohorts.

Our findings are consistent with most of the existing literature including case reports, case series, and smaller studies suggesting similar short- and medium-term survival rates for recipients of COVID-19+ donor heart allografts compared to COVID-19− donor heart allografts [16,17]. In a case series of six recipients who received organs from COVID-19+ donors, three heart recipients had 100% 30-day survival, no acute rejection, and there was no transmission of COVID-19 to the recipient or healthcare personnel [18]. A case series of 12 patients who received COVID-19+ heart transplants by Eichenberger et al. demonstrated a 92% overall survival at a mean follow-up of 173 days among the heart recipients, with no unexpected acute rejection, and no COVID-19 transmission to recipients or healthcare personnel [19]. Mullan et al. analyzed the United Network for Organ Sharing database through 1 December 2021, and identified 32 recipients of heart allografts from COVID-19+ donors and compared them to recipients of COVID-19− donors and found no difference in 30 day or 1 year survival [20]. Similarly, Schold et al. used the Scientific Registry of Transplant Recipients with data up to 31 August 2021 to analyze 62 recipients of COVID-19+ donor heart allografts and found no difference in 6-month survival compared to those who received COVID-19− donor heart allografts [21]. Two additional studies explored this question utilizing the OPTN database, both reporting comparable incidence of short-term mortality and acute rejection for recipients of COVID-19+ donor hearts [8,9]. Compared to these previous studies, our propensity-matched study, featuring a significantly larger sample size, adds to the growing body of literature with a longer 1 year follow up results and corroborates prior findings.

The findings of our sub-group analysis of recipients who received heart allografts from donors with an active COVID-19 infection were concordant with the principal findings of no difference in overall survival. This might be expected given the lack of COVID-19 transmission between donors and recipients based on the existing literature [18,19,22,23], and therefore donor heart allografts deemed suitable for transplantation had withstood the inflammatory effects of COVID-19 in the short-term period. It is possible that donor heart allografts which were ultimately not accepted due to poor organ quality or performance may have been impacted by the systemic inflammatory effects of COVID-19; it would be beneficial to be able to determine the severity of the COVID-19 infection as it pertains to the donor’s systemic inflammatory status prior to donation to assess for correlations with heart allograft performance given the effects of COVID-19 on myocardial and endothelial cell injury [24,25,26,27,28].

A recent study by Madan et al. also looked at propensity score-matched outcomes, primarily 6 months and 1 year survival, in recipients of COVID-19+ donor hearts using the OPTN dataset from January 2020 to June 2022 with follow up until September 2022 [29]. Contrary to our findings, their study reports increased mortality in recipients of hearts from donors with active COVID-19. However, it reports similar mortality in recently recovered COVID-19 donors (previously tested positive followed by a subsequent negative test closest to procurement) as compared to COVID-19− donors in unadjusted and adjusted analyses. Additionally, recipients from COVID-19+ donors overall in the unmatched cohort had similar mortality to recipients from COVID-19− donors in the unadjusted analysis, which is in line with our study findings. However, opposed to our findings, there was increased mortality in the adjusted analysis. For secondary outcomes, like our findings, the study reports similar outcomes of pacemaker placement, dialysis, and stroke between the groups. Some of the drivers of this difference in the findings from our study could be due to the cohort selection in this study that includes data within 6 months from the date of OPTN data harvest which, as previously described, leads to informative censoring and over-representation of adverse outcomes in unbalanced ways [30]. The difference in our results could also be attributed to differences in building the analytic cohort, defining COVID-19 positivity in our cohort by including those who tested positive in final antigen tests in addition to those who tested positive in final NATs, and the study time frame.

Although there was no difference in survival, recipients of COVID-19+ donor heart allografts were less likely to experience acute rejection prior to discharge compared to recipients of COVID-19− donor heart allografts. This finding has not been previously described and is intuitively perplexing. One possible explanation is that patients who experienced severe COVID-19 due to a robust immune-mediated response may not have been considered eligible for heart donation. As a result, this may have excluded donors who might have had highly immunogenic hearts from the COVID-19+ group. Given the strength of this association, further investigation is warranted.

This study has several limitations. The retrospective observational study design does not permit causal inference, may not account for all measured confounders, and cannot account for unmeasured confounding. This study reports on 316 COVID-19+ donor heart allografts, which is a relatively small number for studying a national experience. As mentioned above, we were unable to account for illness severity related to COVID-19. Furthermore, we were unable to account for COVID-19 vaccination status, which will be an important consideration in future work, and we do not have data regarding donor treatment for COVID-19 or post-transplant management of recipients regarding empiric COVID-19 treatment. Finally, we could not assess COVID-19 transmission to procurement personnel and healthcare staff, which is an important concern.

## 5. Conclusions

Our analysis of the OPTN national database demonstrates equivalent survival for up to 1 year post transplant, comparing recipients of heart allografts from donors with a recent history of COVID-19 to those without. We also demonstrated that recipients who received heart allografts from COVID-19+ donors were less likely to experience acute rejection, which requires further investigation. These data should encourage increased utilization of heart allografts from COVID-19+ donors. Further analyses are needed to determine if longer-term outcomes are equivalent between groups.

## Figures and Tables

**Table 1:** Baseline demographic and pre-transplant characteristics for donor and recipients in unmatched cohort.

	Overall (*n* = 7211)	COVID-19+ Donor Heart Recipients (*n* = 316)	COVID-19− Donor Heart Recipients (*n* = 6895)	*p* Value	Missing
RECIPIENT					
Age, Years †	57 (46–64)	55 (44–63)	57 (46–64)	0.11	0
Sex, Male	5268 (73.1%)	241 (76.3%)	5027 (72.9%)	0.19	0
Ethnicity				0.74	0
White	4357 (60.4%)	187 (59.2%)	4170 (60.5%)		
Black	1749 (24.3%)	76 (24.1%)	1673 (24.3%)		
Hispanic	735 (10.2%)	38 (12.0%)	697 (10.1%)		
Other	370 (5.1%)	15 (4.7%)	355 (5.1%)		
Body Mass Index, kg/m^2^	27.5 (24.1–31.3)	27.6 (24.2–32.2)	27.5 (24.1–31.3)	0.32	2
History of Diabetes	2072 (28.7%)	95 (30.1%)	1977 (28.7%)	0.60	4
History of Smoking	2936 (40.7%)	136 (43.0%)	2800 (40.6%)	0.41	0
Dialysis Prior to Transplant	171 (2.4%)	6 (1.9%)	165 (2.4%)	0.57	13
ABO Grouping				0.02 *	0
A	2806 (38.9%)	116 (36.7%)	2690 (39.0%)		
AB	352 (4.9%)	9 (2.8%)	343 (5.0%)		
B	1094 (15.2%)	38 (12.0%)	1056 (15.3%)		
O	2959 (41.0%)	153 (48.4%)	2806 (40.7%)		
Baseline Diagnosis				0.47	0
Non-ischemic Cardiomyopathy	5171 (71.7%)	236 (74.7%)	4935 (71.6%)		
Ischemic Cardiomyopathy	1816 (25.2%)	72 (22.8%)	1744 (25.3%)		
Congenital Heart Disease	224 (3.1%)	8 (2.5%)	216 (3.1%)		
Hospitalization Status at Transplant				0.29	1
Intensive Care	3922 (54.4%)	156 (49.4%)	3766 (54.6%)		
Hospitalized	971 (13.5%)	44 (13.9%)	927 (13.4%)		
Not Hospitalized	2317 (32.1%)	116 (36.7%)	2201 (31.9%)		
On Ventilator at Transplant	158 (2.2%)	5 (1.6%)	153 (2.2%)	0.45	0
On Inotropes at Transplant	2821 (39.1%)	121 (38.3%)	2700 (39.2%)	0.76	0
On ECMO at Transplant	454 (6.3%)	17 (5.4%)	437 (6.3%)	0.49	0
Mean Pulmonary Artery Pressure, mmHg	26.0 (19.0–34.0)	26.0 (19.0–34.5)	26.0 (19.0–34.0)	0.92	250
History of Cardiac Surgery	3020 (41.9%)	141 (44.6%)	2879 (41.8%)	0.32	4
Waiting List Status at Transplant				0.18	0
1	717 (9.9%)	27 (8.5%)	690 (10.0%)		
2	3536 (49.0%)	150 (47.5%)	3386 (49.1%)		
3	1101 (15.3%)	48 (15.2%)	1053 (15.3%)		
4	1437 (19.9%)	79 (25.0%)	1358 (19.7%)		
5	6 (0.1%)	0 (0.0%)	6 (0.1%)		
6	414 (5.7%)	12 (3.8%)	402 (5.8%)		
DONOR					
Donor Age, Years	32 (25–39)	30 (23–37)	32 (25–40)	0.002 **	0
Donor Sex, Male	5234 (72.6%)	248 (78.5%)	4986 (72.3%)	0.02 *	0
Recipient–Donor Sex Mismatch	1448 (20.1%)	57 (18.0%)	1391 (20.2%)	0.35	0
History of Diabetes	302 (4.3%)	16 (5.2%)	286 (4.2%)	0.41	107
History of >20 Pack-Year Smoking	874 (12.5%)	40 (13.2%)	834 (12.5%)	0.73	230
Cocaine Use	1966 (28.0%)	85 (27.6%)	1881 (28.0%)	0.87	195
ABO Match Level				0.32	0
Identical	6181 (85.7%)	280 (88.6%)	5901 (85.6%)		
Compatible	1029 (14.3%)	36 (11.4%)	993 (14.4%)		
Predicted Heart Mass Ratio < 0.86	829 (11.5%)	35 (11.1%)	794 (11.5%)	0.81	2
Donor Cause of Death				0.02 *	0
Anoxia	3335 (46.2%)	150 (47.5%)	3185 (46.2%)		
Stroke	871 (12.1%)	23 (7.3%)	848 (12.3%)		
Head Trauma	2832 (39.3%)	131 (41.5%)	2701 (39.2%)		
Other	173 (2.4%)	12 (3.8%)	161 (2.3%)		
Donation After Circulatory Death	465 (6.4%)	26 (8.2%)	439 (6.4%)	0.19	0
Ischemic Time, Hours	3.5 (2.9–4.0)	3.5 (2.9–4.1)	3.5 (2.9–4.0)	0.87	15

ECMO, extracorporeal membrane oxygenation; SMD, standardized mean difference. † All continuous variables are presented as median (IQR); *p* values * < 0.05 ** < 0.01.

**Table 2:** Baseline demographic and pre-transplant characteristics for donor and recipients in the matched cohort.

	Total (*n*= 1160)	COVID-19+ Donor Heart Recipients (*n* = 290)	COVID-19− Donor Heart Recipients (*n* = 870)	SMD
RECIPIENT				
Age, Years †	56 (44–63)	55 (44–63)	56 (44–63)	0.03
Sex, Male	876 (75.5%)	220 (75.9%)	656 (75.4%)	0.01
Ethnicity				0.01
White	681 (58.7%)	171 (59.0%)	510 (58.6%)	
Black	291 (25.1%)	68 (23.4%)	223 (25.6%)	
Hispanic	117 (10.1%)	37 (12.8%)	80 (9.2%)	
Other	71 (6.1%)	14 (4.8%)	57 (6.6%)	
Body Mass Index, kg/m^2^	27.7 (24.3–31.8)	27.5 (24.2–32.3)	27.8 (24.4–31.6)	0.02
History of Diabetes	353 (30.4%)	90 (31.0%)	263 (30.2%)	0.02
History of Smoking	492 (42.4%)	122 (42.1%)	370 (42.5%)	0.01
Dialysis Prior to Transplant	17 (1.5%)	5 (1.7%)	12 (1.4%)	0.03
ABO grouping				0.12
A	456 (39.3%)	108 (37.2%)	348 (40.0%)	
AB	39 (3.4%)	6 (2.1%)	33 (3.8%)	
B	184 (15.9%)	35 (12.1%)	149 (17.1%)	
O	481 (41.5%)	141 (48.6%)	340 (39.1%)	
Baseline diagnosis				0.09
Non-ischemic Cardiomyopathy	873 (75.3%)	219 (75.5%)	654 (75.2%)	
Ischemic Cardiomyopathy	261 (22.5%)	64 (22.1%)	197 (22.6%)	
Congenital Heart Disease	26 (2.2%)	7 (2.4%)	19 (2.2%)	
Hospitalization Status at Transplant				0.01
Intensive Care	574 (49.5%)	146 (50.3%)	428 (49.2%)	
Hospitalized	175 (15.1%)	41 (14.1%)	134 (15.4%)	
Not Hospitalized	411 (35.4%)	103 (35.5%)	308 (35.4%)	
On Ventilator at Transplant	23 (2.0%)	5 (1.7%)	18 (2.1%)	0.03
On Inotropes at Transplant	471 (40.6%)	112 (38.6%)	359 (41.3%)	0.05
On ECMO at Transplant	67 (5.8%)	16 (5.5%)	51 (5.9%)	0.02
Mean Pulmonary Artery Pressure, mmHg	27.0 (20.0–35.0)	27.0 (19.0–35.0)	27.0 (20.0–35.0)	0.05
History of Cardiac Surgery	503 (43.4%)	126 (43.4%)	377 (43.3%)	0.00
Waiting List Status at Transplant				0.03
1	112 (9.7%)	24 (8.3%)	88 (10.1%)	
2	567 (48.9%)	142 (49.0%)	425 (48.9%)	
3	187 (16.1%)	45 (15.5%)	142 (16.3%)	
4	244 (21.0%)	69 (23.8%)	175 (20.1%)	
5	1 (0.1%)	0 (0.0%)	1 (0.1%)	
6	49 (4.2%)	10 (3.4%)	39 (4.5%)	
DONOR				
Donor Age, Years	30 (22–37)	30 (23–37)	30 (22–37)	0.00
Donor Sex, Male	861 (74.2%)	226 (77.9%)	635 (73.0%)	0.12
Recipient–Donor Sex Mismatch	203 (17.5%)	52 (17.9%)	151 (17.4%)	0.02
History of Diabetes	49 (4.2%)	16 (5.6%)	33 (3.8%)	0.08
History of >20 Pack-Year Smoking	142 (12.2%)	39 (13.4%)	103 (11.8%)	0.05
Cocaine Use	324 (27.9%)	80 (27.6%)	244 (28.0%)	0.01
ABO Match Level				0.07
Identical	1008 (86.9%)	257 (88.6%)	751 (86.3%)	
Compatible	152 (13.1%)	33 (11.4%)	119 (13.7%)	
Predicted Heart Mass Ratio < 0.86	134 (11.6%)	33 (11.4%)	101 (11.6%)	0.07
Donor Cause of Death				0.02
Anoxia	508 (43.8%)	132 (45.5%)	376 (43.2%)	
Stroke	106 (9.1%)	22 (7.6%)	84 (9.7%)	
Head Trauma	505 (43.5%)	124 (42.8%)	381 (43.8%)	
Other	41 (3.5%)	12 (4.1%)	29 (3.3%)	
Donation After Circulatory Death	92 (7.9%)	23 (7.9%)	69 (7.9%)	0.00
Ischemic Time, Hours	3.5 (2.9–4.0)	3.5 (2.9–4.0)	3.5 (2.9–4.0)	0.05

ECMO, extracorporeal membrane oxygenation; SMD, standardized mean difference. † All continuous variables are presented as median (IQR).

## Data Availability

All data used in this study are available by request from the Organ Procurement and Transplantation Network.

## Abbreviations

COVID-19	Coronavirus Disease 2019
ECMO	Extracorporeal Membrane Oxygenation
NAT	Nucleic Acid Amplification Test
OPTN	Organ Procurement and Transplantation Network
PCR	Polymerase Chain Reaction
PH	Proportional Hazards
SARS-CoV-2	Severe Acute Respiratory Syndrome Coronavirus 2
